# Human Dental Pulp Stem Cells Modulate Cytokine Production *in vitro* by Peripheral Blood Mononuclear Cells From Coronavirus Disease 2019 Patients

**DOI:** 10.3389/fcell.2020.609204

**Published:** 2021-02-05

**Authors:** Stefania Croci, Martina Bonacini, Giovanni Dolci, Marco Massari, Nicola Facciolongo, Elisa Pignatti, Alessandra Pisciotta, Gianluca Carnevale, Aurelio Negro, Giulia Cassone, Francesco Muratore, Lucia Belloni, Alessandro Zerbini, Carlo Salvarani

**Affiliations:** ^1^Clinical Immunology, Allergy and Advanced Biotechnologies Unit, Azienda Unità Sanitaria Locale-IRCCS di Reggio Emilia, Reggio Emilia, Italy; ^2^Infectious Disease Unit, Azienda Unità Sanitaria Locale-IRCCS di Reggio Emilia, Reggio Emilia, Italy; ^3^Pulmonology Unit, Azienda Unità Sanitaria Locale-IRCCS di Reggio Emilia, Reggio Emilia, Italy; ^4^Department of Surgery, Medicine Dentistry and Morphological Sciences with Interest in Transplant, University of Modena and Reggio Emilia, Modena, Italy; ^5^Internal Medicine and Secondary Hypertension Center, Azienda Unità Sanitaria Locale-IRCCS di Reggio Emilia, Reggio Emilia, Italy; ^6^PhD Program in Clinical and Experimental Medicine, University of Modena and Reggio Emilia, Modena, Italy; ^7^Rheumatology Unit, Azienda Unità Sanitaria Locale-IRCCS di Reggio Emilia, Reggio Emilia, Italy

**Keywords:** COVID-19, SARS-CoV-2, mesenchymal stem cells, dental pulp stem cells, cytokine, interleukin, immunomodulation

## Abstract

A subset of patients infected with severe acute respiratory syndrome coronavirus 2 (SARS-CoV-2) developed a condition of hyper-inflammation, which can cause multi-organ damage and the more severe forms of coronavirus disease 2019 (COVID-19). Mesenchymal stem cells (MSCs) can promote tissue regeneration and modulate immune responses and, thus, have the rational requirements to be used to counteract SARS-CoV-2-induced pneumonia and hyper-inflammation. The aim of the present study was to gain insight into possible mechanisms of action of MSCs obtained from human dental pulp [dental pulp stem cells (DPSCs)] in COVID-19 patients. We investigated the concentrations of 18 cytokines in supernatants of peripheral blood mononuclear cells (PBMCs) obtained from COVID-19 patients cultured *in vitro* alone and in contact with DPSCs. The modulation of cytokines in PBMCs was confirmed by real-time PCR. IL-6 was the sole cytokine detected in supernatants of DPSCs. In resting conditions, co-culture increased IL-1β, IL-2, IL-5, IL-6, IL-10, IL-18, TNFα, and granulocyte macrophage colony-stimulating factor (GM-CSF) levels. When PBMCs were activated with anti-CD3/CD28 antibody-coated beads, co-culture increased IL-6 and GM-CSF, whereas it decreased IFNγ, TNFα, IL-2, IL-5, IL-9, IL-10, IL-12 (p70), IL-17A, IL-18, IL-21, IL-23, and IL-27 levels. Concentrations of IL-1β, IL-4, IL-13, and IL-22 were not affected. The comparison of cytokine concentrations in supernatants of PBMCs from COVID-19 patients vs. healthy subjects revealed lower concentrations of IL-10 and higher concentrations of IL-18 in supernatants of CD3/CD28-activated PBMCs from COVID-19 patients. Results are explorative but indicate that DPSCs can modulate the production of cytokines deregulated in COVID-19 patients, supporting their potential use in COVID-19.

## Introduction

Severe acute respiratory syndrome coronavirus 2 (SARS-CoV-2) is the agent responsible for the coronavirus disease 2019 (COVID-19). It is a betacoronavirus, endowed with envelope and single-stranded RNA genome. SARS-CoV-2 can infect angiotensin I-converting enzyme 2 (ACE-2) receptor-expressing cells, mainly lung epithelial cells and capillary endothelial cells. Following SARS-CoV-2 infection, the immune system is activated to clear the virus. In the majority of the patients, infected cells are eliminated and viruses are inactivated with minimal inflammation and lung damage. Instead, in a subset of patients, a condition of hyper-inflammation characterized by the production of several cytokines and excessive immune cell infiltration into tissues occur, causing multi-organ damage and severe COVID-19 (Tay et al., 2020). Evidences on the pathogenic role of host immune responses in SARS-CoV-2 infection represent the rationale for testing the efficacy of anti-inflammatory and immunosuppressive drugs/products in COVID-19 patients who show hyper-inflammation (Calabrese, 2020; Rizk et al., 2020).

Mesenchymal stem cells (MSCs) are non-hematopoietic multipotent progenitor cells capable of self-renewal and differentiation into various mesenchymal tissues: chondrocytes, osteoblasts, and adipocytes. MSCs can favor tissue repair and remodeling and can modulate innate and adaptive immune responses through soluble factors and direct cell–cell contact (Glenn and Whartenby, 2014; Uccelli and de Rosbo, 2015; Ansboro et al., 2017). The source of human MSCs can impact their immunoregulatory and regenerative abilities. In the present project, we employed stem cells isolated from human dental pulp [dental pulp stem cells (DPSCs)], obtained following routine dental procedures (Gronthos et al., 2002; Pierdomenico et al., 2005; Pisciotta et al., 2015). DPSCs are characterized by a high proliferation rate and low immunogenicity, allowing allogeneic administrations. Moreover, they are able to differentiate into the osteogenic, chondrogenic, adipogenic, myogenic, and neural lineages due to their embryological origin from the neural crest (Pisciotta et al., 2020b). Finally, DPSCs own immunomodulatory properties, e.g., through the expression of FasL and the release of soluble factors (Demircan et al., 2011; Zhao et al., 2012; Spagnuolo et al., 2018; Pisciotta et al., 2020a; Zayed and Iohara, 2020).

Relying on MSC immune regulatory and tissue repair properties, the safety and clinical efficacy of MSC treatment have been tested in several autoimmune and inflammatory diseases (Regmi et al., 2019). Besides, MSC use has been suggested as a rational possibility to counteract SARS-CoV-2-induced pneumonia and hyper-inflammation (Gentile and Sterodimas, 2020; Golchin et al., 2020; Liu S. et al., 2020; Rogers et al., 2020; Saldanha-Araujo et al., 2020). This strategy is supported by 70 position articles or original research articles on MSCs in COVID-19 listed in PubMed. Moreover, there are 65 clinical studies evaluating the efficacy and the safety of MSC or MSC-derived products (e.g., extracellular vesicles) administration in COVID-19 patients in ClinicalTrials.gov up to 9th September 2020. Following intravenous administration, MSCs mainly accumulate in the lungs, which can be favorable for treatment of pulmonary diseases. The main sources of human MSCs are the bone marrow, adipose tissue, and umbilical cord blood; but there are also a couple of clinical studies that rely on DPSCs (Ye et al., 2020; Zayed and Iohara, 2020).

Published results on MSC therapy in COVID-19 patients are few but promising, thus fostering further investigations in this field. Leng et al. (2020) reported that MSC intravenous administration improved pulmonary function and symptoms in seven patients with COVID-19 pneumonia, particularly in patients with more severe disease. MSC administration was associated with an increase in peripheral lymphocytes, regulatory T cells, dendritic cells, and IL-10 and a decrease in CXCR3+ T and NK cells, TNFα, and C-reactive protein (CRP). Following the administration of MSC from the umbilical cord and menstrual blood in COVID-19 patients, laboratory parameters, oxygenation, and pulmonary function improved (Guo et al., 2020; Liang et al., 2020; Meng et al., 2020; Shu et al., 2020; Tang et al., 2020; Zhang et al., 2020). Different doses of MSCs were intravenously injected in the patients in the various trials with different schedules: 1 × 10^6^ or 2 × 10^6^ cells per kilogram of weight once (Guo et al., 2020; Leng et al., 2020; Shu et al., 2020; Zhang et al., 2020); 1 × 10^6^ cells per kilogram of weight three times (Tang et al., 2020); and 3 × 10^7^ or 5 × 10^7^ cells three times (Liang et al., 2020; Meng et al., 2020). Finally, exosomes derived from allogeneic bone marrow MSCs have been used to treat 24 severe COVID-19 patients by intravenous administration: 71% of the patient recovered and were discharged from the hospital (Sengupta et al., 2020). Overall, no adverse side effects have been observed in patients treated with MSCs and MSC-derived products.

The aim of the present study was to gain insight into possible mechanisms of actions of MSCs in COVID-19 patients, investigating whether DPSCs can modulate cytokine production *in vitro* by peripheral blood mononuclear cells (PBMCs) from patients with COVID-19 pneumonia and hyper-inflammation.

## Materials and Methods

### Patients

Nine patients with COVID-19 pneumonia showing an increased inflammatory response were included in the study. Patients were positive for SARS-CoV-2 by real-time PCR on respiratory tract swabs, showed the typical findings of COVID-19 pneumonia diagnosed by chest high-resolution computed tomography and required hospitalization at the Azienda Unità Sanitaria Locale-IRCCS at Reggio Emilia (Italy). Moreover, all patients had an increased inflammatory response as showed by elevated serum levels of CRP and IL-6 [median CRP = 8.6 mg/dl, interquartile range (IQR): 7.2–15.8; median IL-6 = 103.8 pg/ml, IQR: 23.3–116.3] and by the presence of temperature >38°C in the last 2 days. Clinical characteristics of the patients are listed in Table 1. CRP levels were measured using ADVIA kit for wr-CRP according to the manufacturer’s instructions (Siemens Healthineers, Erlangen, Germany). The upper limit of the normal reference range was 0.5 mg/dl. Serum IL-6 concentrations were measured using the IVD Elecsys IL-6 assay according to the manufacturer’s instructions (Roche Diagnostics, Basel, Switzerland). IL-6 levels < 7 pg/ml (95° percentile) were considered as normal. At the moment of blood withdrawal, therapy with corticosteroids and biologic drugs constituted exclusion criteria. Patients requiring invasive mechanical ventilation and intensive care were excluded as well as pregnant women and breastfeeding mothers.

**TABLE 1:** Characteristics of the COVID-19 patients whose PBMCs were used for the *in vitro* assays.

Patient ID	Age (years)	CRP (mg/dl)	IL-6 (pg/ml)	Lymphocytes/μl	Monocytes/μl	Neutrophils/μl
1	75	8.6	66.3	700	140	4,800
2	86	16.0	103.8	460	290	4,850
3	68	7.6	116.6	610	140	3,590
4	54	6.7	27.9	660	190	4,630
5	80	10.4	104.5	920	420	6,420
6	42	7.7	18.6	590	240	2,850
7	69	15.6	299.1	600	230	5,230
8	66	15.9	115.9	1,010	430	7,880
9	59	2.3	11.7	640	360	3,550

*Normal ranges: CRP < 0.5 mg/dl; IL-6 < 7 pg/ml; lymphocytes, 800–4,000/μl; monocytes, 80–1,000/μl; neutrophils, 1,600–7,500/μl. All the patients had temperature >38°C in the last 2 days and had pneumonia diagnosed by chest high-resolution computed tomography. PBMCs, peripheral blood mononuclear cells; CRP, C-reactive protein.*

The study was approved by the local ethics committee (“Comitato Etico dell’Area Vasta Emilia Nord,” protocol number 2020/0062210, study 594/2020/TESS/AUSLRE). It was performed in compliance with the Declaration of Helsinki. Written informed consent was obtained from all patients.

### Healthy Subjects

Healthy subjects (*n* = 9) were recruited within the study entitled “Dental pulp stem cells: *in vitro* studies on the modulation of the immune responses in autoimmune diseases” approved on 11 October 2017 by the local ethics committee, with protocol number AUSLRE 2017/0100677. Written informed consent was obtained from all subjects.

### Dental Pulp Stem Cells

Dental Pulp Stem Cells were provided as liquid nitrogen frozen cells by Prof Gianluca Carnevale. Human DPSCs were isolated from third molars of adult subjects undergoing routine dental extraction (*n* = 5, age 18–35 years). Briefly, after enzymatic digestion and immune selection against the surface antigens STRO-1 and c-Kit, cells were seeded and allowed to grow. Before reaching confluence, they were detached, characterized by flow cytometry for the expression of the typical MSC markers (Dominici et al., 2006), and stored frozen at passage 1 in medium containing 90% heat-inactivated fetal bovine serum (FBS; Gibco, Thermo Fisher Scientific, Monza, Italy) 10% dimethyl sulfoxide (Sigma-Aldrich, Milan, Italy) in liquid nitrogen until use. DPSCs expressed HLA-ABC and the typical MSC markers CD73, CD90, and CD105, barely expressed CD34, while they did not express CD45 and HLA-DP-DQ-DR (Pisciotta et al., 2015, 2020a).

### Isolation of Peripheral Blood Mononuclear Cells

Peripheral Blood Mononuclear Cells were isolated by Histopaque-1077 density gradient centrifugation (Sigma-Aldrich, Milan, Italy) and stored frozen in liquid nitrogen in 90% heat-inactivated FBS 10% dimethyl sulfoxide until use. Biosafety level 2 procedures were applied when working with biological samples (Cossarizza et al., 2020). To provide barriers between samples and personnel, scientists wore personal protective equipment. Samples were handled in certified class II vertical laminar-flow biological safety cabinets. Centrifuges had safety cups to prevent aerosols. Work surfaces and equipment were decontaminated using disinfectant solutions with proven activity against enveloped RNA viruses. All liquid and solid wastes were decontaminated before disposal.

### Co-culture Between Dental Pulp Stem Cells and Peripheral Blood Mononuclear Cells

Dental Pulp Stem Cells obtained from a 24-year-old donor were thawed, cultured to be expanded, and then seeded at 50,000 cells/well in 24-well plates in MEM alpha medium + 10% heat-inactivated FBS and allowed to adhere overnight. The following day, PBMCs were thawed, suspended in RPMI + 10% heat-inactivated FBS, and counted with a Burker hemocytometer with trypan blue dye to evaluate and exclude dead cells. The medium was changed, and PBMCs were added to DPSCs at 10:1 effector:target ratio both resting (unstimulated) and stimulated with anti-CD3/CD28 beads (1:1 cell:bead ratio, dynabeads^TM^ human T-activator CD3/CD28) in 500 μl of RPMI + 10% heat-inactivated FBS. Cell culture media and dynabeads were purchased from Thermo Fisher Scientific. DPSCs, resting PBMCs, and CD3/CD28-stimulated PBMCs cultured alone were included as controls. Supernatants were collected after 48 h, centrifuged at 10,000 × *g* for 1 min to remove PBMCs and stored at −80°C until use. PBMC pellets were also stored at −80°C from five patients. We performed two assays: the first included samples from patient IDs 1, 2, 3, 4, and 5, and the second included samples from patient IDs 6, 7, 8, and 9. DPSCs at passage 3 were used.

### Quantification of Cytokines in Culture Supernatants

Eighteen cytokines were quantified in culture supernatants with the Procarta Plex Th1, Th2, Th9, Th17, Th22, Treg cytokine panel (eBioscience, Thermo Fisher Scientific) following the manufacturer’s instructions. Thawed supernatants were centrifuged at 10,000 × *g* for 1 min at 4°C prior to plating. Eight serial dilutions of cytokine standards plus blank (RPMI + 10% FBS) were included. Data were obtained with the Bio-Plex MAGPIX^TM^ multiplex reader and analyzed with the Bio-Plex^TM^ Manager software. Standard curves were calculated with the five-parameter logistic equation regression method. The lower limit of detection of the cytokines were as follows: IL-1β, 0.9 pg/ml; IL-2, 4.8 pg/ml; IL-4, 2.8 pg/ml; IL-5, 2.5 pg/ml; IL-6, 5.1 pg/ml; IL-9, 4.1 pg/ml; IL-10, 1.1 pg/ml; IL-12 (p70), 1.9 pg/ml; IL-13, 0.7 pg/ml; IL-17A, 0.5 pg/ml; IL-18, 4.3 pg/ml; IL-21, 1.2 pg/ml; IL-22, 12.1 pg/ml; IL-23, 35.0 pg/ml; IL-27, 8.9 pg/ml; IFNγ, 4.5 pg/ml; TNFα, 3.0 pg/ml; and granulocyte macrophage colony-stimulating factor (GM-CSF), 3.5 pg/ml.

### Gene Expression Profiling

RNA was extracted from the available samples of PBMCs stored frozen at −80°C as cell pellets at the moment of supernatant collection (*n* = 5, corresponding to patient IDs 1, 2, 3, 6, and 7). RNA was extracted with the Quick-DNA/RNA Microprep Plus kit (Zymo Research) and quantified with the NanoDrop instrument (Thermo Scientific). 62.5 ng of RNA was reverse transcribed with the PrimeScript RT Reagent Kit with gDNA Eraser (Takara) in a total volume of 20 μl. Real-time PCR was performed with the SYBR Premix Ex Taq II (Tli RNaseH Plus) containing the ROX Reference Dye (Takara) and QuantiTect primer assays (Qiagen): GAPDH (QT01192646), POLR2A (QT00033264), ACTB (QT01680476), IL-6 (QT00083720), IL-17 (QT00009233), or forward and reverse primers reported in the manuscript by Watanabe et al. (2018) (IL-21) and Conway et al. (2018) (IFNγ, TNFα, IL-10, IL-12p35, IL-12p40, and IL-23p19) at 0.4-μM concentrations. Geometric means of the cycle threshold (Ct) values of GAPDH, POLR2A, and ACTB were used to normalize gene expression data in each sample. Gene expression levels were calculated as 2^–ΔCt^. We investigated gene expression of the cytokines, which resulted modulated in supernatants from all the samples (TNFα, IL-12, IL-21, IL-23, and IL-6) and/or are relevant for COVID-19 (IL-10, IL-17, and IFNγ). It is to be noted that IL-12 and IL-23 are heterodimeric proteins and share the p40 subunit. IL-12 is encoded by two separate genes: IL-12A (p35) and IL-12B (p40). IL-23 is also encoded by two separate genes: IL-12B (p40) and IL23A (p19).

### Statistics

Data were analyzed with GraphPad Prism 6 using nonparametric tests: Wilcoxon-matched paired test (untreated vs. DPSC-treated PBMCs) and Mann–Whitney test (PBMCs from COVID-19 patients vs. controls). Fold changes were analyzed with Wilcoxon signed-rank test vs. a theoretical value = 1. *Post hoc* analysis was performed with G^∗^Power software version 3.1.9.4 using Wilcoxon signed-rank test (one sample case), two tails, given α = 0.05, total sample size = 9, and effect size “*d*” calculated based on the mean fold changes (DPSC-treated PBMCs/untreated PBMCs) and standard deviations obtained for the levels of each cytokine in conditioned media.

## Results

### Effects of Dental Pulp Stem Cells on Resting Peripheral Blood Mononuclear Cells From Coronavirus Disease 2019 Patients

**FIGURE 1:**
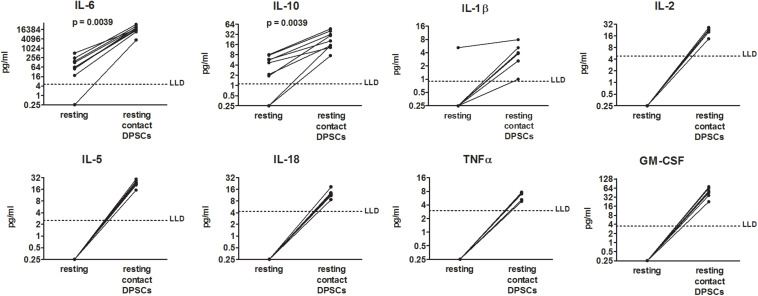
Effects of dental pulp stem cells (DPSCs) on peripheral blood mononuclear cells (PBMCs) from coronavirus disease 2019 (COVID-19) patients in resting condition. PBMCs from nine COVID-19 patients were cultured alone and in contact with DPSCs at 10:1 effector:target ratio. Concentrations of 18 cytokines (pg/ml) were determined in 48-h conditioned media by means of a multiplex bead-based assay. Dotted lines indicate lower limits of detection (LLD). When cytokine concentrations were lower than the limits of detection or not detected, a value = 0.25 was arbitrarily assigned. Data were analyzed by Wilcoxon-matched paired test.

To determine the effects of DPSCs on PBMCs from COVID-19 patients, PBMCs were added to DPSCs monolayer at 10:1 ratio and then co-cultured for 48 h. Concentrations of 18 cytokines were measured in conditioned media. Among the investigated cytokines, DPSCs cultured alone showed only the production of IL-6 in supernatants (median = 74.9 pg/ml, IQR: 58.1–133.5 pg/ml). IL-6 and IL-10 were detected in supernatants from resting PBMCs from COVID-19 patients and were increased 225- and 6-fold, respectively, after co-culture (*p* = 0.0039 by Wilcoxon-matched pairs test) (Figure 1). Moreover IL-1β, IL-2, IL-5, IL-18, TNFα, and GM-CSF became detectable in all the samples following co-culture between resting PBMCs from COVID-19 patients and DPSCs (Figure 1). Raw data are reported in Supplementary Table 1. mRNA profiling of PBMCs collected by centrifugation of culture supernatants confirmed the up-regulation of IL-6, while IL-10 and TNFα appeared unchanged following co-culture (Supplementary Figures 1A,C, and Ct values listed in Supplementary Table 2).

### Effects of Dental Pulp Stem Cells on CD3/CD28-Stimulated Peripheral Blood Mononuclear Cells From Coronavirus Disease 2019 Patients

**FIGURE 2:**
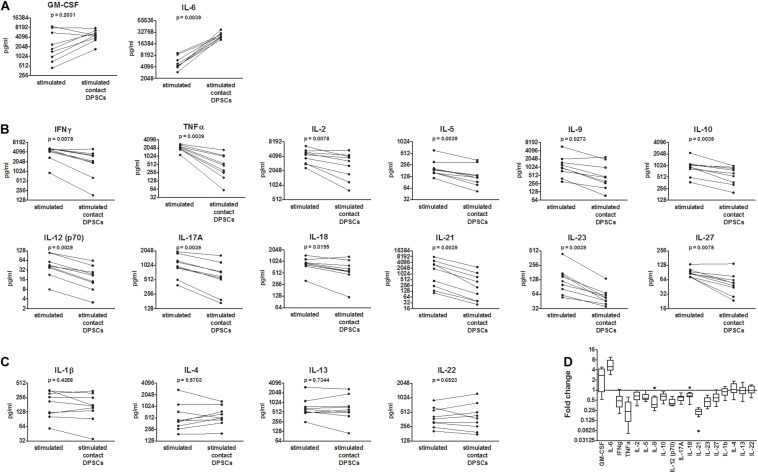
Effects of dental pulp stem cells (DPSCs) on CD3/CD28-stimulated peripheral blood mononuclear cells (PBMCs) from coronavirus disease 2019 (COVID-19) patients. PBMCs from nine COVID-19 patients were activated with anti-CD3/CD28-coated beads and then cultured alone and in contact with DPSCs at 10:1 effector:target ratio. Concentrations of 18 cytokines were determined in 48 h conditioned media by means of a multiplex bead-based assay (pg/ml). **(A)** Cytokines up-regulated following co-culture. **(B)** Cytokines down-regulated following co-culture. **(C)** Cytokines not affected by co-culture. Data were analyzed by Wilcoxon-matched paired test. *p* values < 0.05 were considered statistically significant. **(D)** Fold changes = cytokine concentrations in supernatants of DPSC-treated PBMC/cytokine concentrations in supernatants of untreated PBMCs shown as Tukey box and whiskers. Wilcoxon signed-rank test vs. a theoretical value = 1 indicated statistically significant fold changes for all the cytokines (*p* < 0.05) with the exception of granulocyte macrophage colony-stimulating factor (GM-CSF) (*p* = 0.0547) and IL-1β, IL-4, IL-13, and IL-22 (non-significant).

To mimic *in vitro* the activation of T lymphocytes that occurs in infected tissues *in vivo*, anti-CD3/CD28 antibody-coated beads were added to PBMCs. PBMC activation led to an increased production of all the investigated cytokines compared with resting PBMCs. Co-culture between activated PBMCs and DPSCs led to a further increase in IL-6 levels in all the samples (median fold change = 5.1, IQR: 4.7–6.2) and GM-CSF in six out of nine samples (Figures 2A,D). The increase in GM-CSF apparently occurred when basal levels were lower than 4,000 pg/ml. On the contrary, concentrations of IFNγ, TNFα, IL-2, IL-5, IL-9, IL-10, IL-12 (p70), IL-17A, IL-18, IL-21, IL-23, and IL-27 were decreased in supernatants after co-culture (Figures 2B,D). In particular, TNFα, IL-12 (p70), IL-21, and IL-23 were the most inhibited cytokines being reduced in all the samples by at least 1.4-fold and showing a median decrease of fivefold, fivefold, 2.5-fold, and twofold respectively. The inhibition of IL-2, IL-10, and IL-18 was less consistent, occurring in six out of the nine samples considering a 1.4-fold change cutoff. Concentrations of IL-1β, IL-4, IL-13, and IL-22 were not affected by co-culture (Figures 2C,D). Raw data are reported in Supplementary Table 1. *Post hoc* analysis using G^∗^Power software revealed an achieved power greater than 0.90 for all the investigated cytokines with the exception of GM-CSF, IL-1β, IL-4, IL-13, and IL-22 (Supplementary Table 3).

mRNA profiling of PBMCs collected by centrifugation of culture supernatants confirmed the up-regulation of IL-6 and the down-regulation of TNFα, IFNγ, IL-10, IL-17, IL-21, and IL-12p40 in CD3/CD28-stimulated PBMCs following co-culture with DPSCs (Supplementary Figure 1B,C, and Ct values listed in Supplementary Table 2).

### Differences Between Peripheral Blood Mononuclear Cells From Coronavirus Disease 2019 Patients and Controls

**FIGURE 3:**
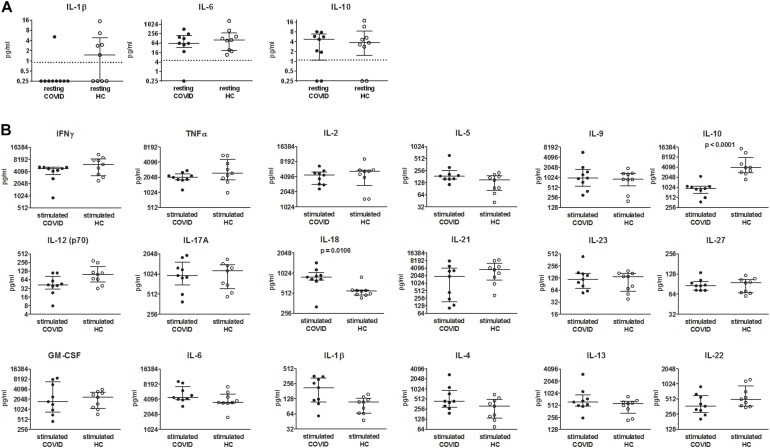
Comparison of cytokine levels in media conditioned by peripheral blood mononuclear cells (PBMCs) from coronavirus disease 2019 (COVID-19) patients and healthy controls. **(A)** PBMCs were cultured for 48 h in RPMI + 10% heat-inactivated fetal bovine serum (FBS). **(B)** PBMCs were cultured for 48 h in RPMI + 10% heat-inactivated FBS in presence of anti-CD3/CD28-coated beads. Concentrations of 18 cytokines were determined by means of a multiplex bead-based assay (pg/ml). When cytokine concentrations were lower than the limits of detection or not detected, a value = 0.25 was arbitrarily assigned. Data were analyzed by Mann–Whitney test. *p* values < 0.05, considered statistically significant, are shown. Lines indicate median with interquartile range. HC, healthy controls. Dotted lines indicate lower limits of detection.

To determine if PBMCs from COVID-19 patients have specific features, cytokine concentrations in 48-h conditioned media were compared between PBMCs from COVID-19 patients and healthy controls both resting and CD3/CD28 activated. In resting conditions, only IL-1β, IL-6, and IL-10 were detected in supernatants and showed similar levels between COVID-19 patients and healthy controls (Figure 3A). Following PBMC activation with anti-CD3/CD28 antibodies, IL-10 showed lower levels, while IL-18 showed higher levels in supernatants of PBMCs from COVID-19 patients. No differences were detected in the other cytokines (Figure 3B). Raw data regarding cytokine concentrations in culture supernatants of PBMCs from healthy controls are reported in Supplementary Table 4.

## Discussion

The present data indicate that DPSC administration might be effective in patients with COVID-19-induced hyper-inflammation, due to the inhibition in the production of several cytokines by activated T lymphocytes, namely, IFNγ, TNFα, IL-2, IL-9, IL-10, IL-17A, IL-18, IL-21, and IL-27. These cytokines have shown higher circulating levels in patients with COVID-19 compared with healthy controls and might thus have a role in disease pathogenesis. Moreover, higher levels of IFNγ TNFα, IL-2, and IL-10 have been associated with COVID-19 severity, further supporting a pathogenic role (Akbari et al., 2020; Chi et al., 2020; Costela-Ruiz et al., 2020; Del Valle et al., 2020; Lucas et al., 2020; Wang et al., 2020). Higher percentages of CD4+ T cells producing IFNγ, TNFα, IL-2, and IL-17A and CD8+ T cells producing IL-2 and IL-17A have been detected by flow cytometry in PBMCs from COVID-19 patients vs. healthy controls after *in vitro* stimulation (De Biasi et al., 2020).

Previous *in vitro* studies on the effects of MSCs on cytokine production by phytohemagglutinin- or CD3/CD28-activated PBMCs from healthy subjects and patients with inflammatory and autoimmune diseases are in agreement with the herein results concerning the reduction in TNFα, IFNγ, IL-2, IL-12, and IL-17, while they are discordant regarding the effects on IL-10. An increase instead of a decrease in the production of IL-10 has been indeed reported by other scientists following co-culture between activated PBMCs and MSCs (Gonzalez-Rey et al., 2010; Demircan et al., 2011; Ozdemir et al., 2016; Yang et al., 2016; Baharlou et al., 2017; Zhou et al., 2018; Ma et al., 2019; Liu Y. et al., 2020).

The present data indicate that contact between DPSCs and PBMCs can lead to a strong increase in IL-6 and GM-CSF. IL-6 can be produced by MSCs themselves, including DPSCs [herein results and Chen et al. (2008); Melief et al. (2013); Kondo et al. (2015), and Park et al. (2019)]. The role of IL-6 in MSCs still needs to be fully clarified, but some data indicate that IL-6 can regulate MSC stemness, differentiation, and immune modulation abilities. There are few published data on IL-6 and GM-CSF levels following co-culture between MSCs and PBMCs. Bertolo et al. (2017) have reported an increase in IL-6 but no changes in GM-CSF in culture supernatants. Demircan et al. (2011) have reported an increase in IL-6 in DPSCs but a decrease in IL-6 in T cells evaluated by flow cytometry. Baharlou et al. (2017) have reported no changes in IL-6. Whether an up-regulation of IL-6 and GM-CSF could be beneficial or harmful for patients with COVID-19 is difficult to predict based on the current knowledge. Indeed, both IL-6 and GM-CSF can have a dual role as proinflammatory and regulatory cytokines, depending on the concentration, target cells, organs, and local microenvironment.

IL-6 is produced during the acute phase of inflammation and can tune the immune system, controlling the transition from acute to chronic inflammation, and can also regulate tissue regeneration and various physiological processes involving endothelial cells, smooth muscle cells, and intestinal epithelial cells (Schaper and Rose-John, 2015). IL-6 can be overexpressed by COVID-19 patients, and higher levels of IL-6 have been shown to be associated with COVID-19 severity and worse prognosis (Costela-Ruiz et al., 2020; Del Valle et al., 2020). Starting from these evidences, IL-6 has been proposed as a promising therapeutic target in COVID-19. Neutralization of IL-6 signaling using anti-IL-6 receptor antibodies (tocilizumab and sarilumab) or anti-IL-6 antibodies (clazakizumab) have been tested in patients with COVID-19 showing hyper-inflammation. The first results deriving from observational studies have appeared promising, indicating that patients with severe COVID-19 pneumonia treated with tocilizumab have more favorable outcomes (Cortegiani et al., 2020; Guaraldi et al., 2020). However, results from two randomized controlled trials, one of them promoted by our institute and supported by the Italian Agency for Medicines (AIFA), have indicated no significant benefit in terms of either mortality or intensive care unit admission in COVID-19 patients treated with tocilizumab as compared with those in the control arm (manuscripts submitted for publication). Therefore, to date, the role of IL-6 in SARS-CoV-2 infection still needs to be defined.

Granulocyte macrophage colony-stimulating factor can promote inflammation inducing the proliferation and differentiation of macrophages and dendritic cells, leading to tissue damage. On the other hand, GM-CSF can have lung-protective effects, favoring alveolar epithelial repair and fostering pulmonary host defense against pathogens. COVID-19 patients have increased levels of GM-CSF in serum/plasma (Costela-Ruiz et al., 2020). Either the administration of recombinant GM-CSF or anti-GM-CSF neutralizing antibody has been suggested as potential therapeutic strategies in different phases of the disease (Lang et al., 2020). Results of clinical trials have not been released yet.

Dental pulp stem cells exerted similar effects on the levels of IL-6 and GM-CSF in culture supernatants by resting vs. CD3/CD28-stimulated PBMCs from COVID-19 patients: up-regulation. Instead, DPSCs exerted opposite effects on the levels of IL-2, IL-5, IL-10, IL-18, and TNFα in culture supernatants by resting vs. CD3/CD28-stimulated PBMCs: up-regulation vs. down-regulation. It is likely that in resting conditions, innate immune cell responses by monocytes and NK cells were mainly detected by the analysis of cytokine levels in culture supernatants, whereas the anti-CD3/CD28 stimulus allowed to highlight mainly T lymphocyte responses. Besides, it is known that high levels of IFNγ and TNFα can drive MSCs toward an immunosuppressive phenotype characterized by the up-regulation of molecules expressed on cell membrane [e.g., programmed cell death ligands 1 and 2 (PD-L1 and PD-L2)] as well as secreted [e.g., indoleamine 2,3-dioxygenase (IDO)] (Carvalho et al., 2019; Noronha et al., 2019; Jiang and Xu, 2020). Following CD3/CD28 stimulation of PBMCs, all the investigated cytokines were highly detected in culture supernatants, including IFNγ and TNFα, which likely modified DPSC phenotype toward a more immunosuppressive one. This could explain the different effects of DPSCs on resting compared with CD3/CD28-activated PBMCs.

Cytokine levels were investigated in conditioned media by a multiplex bead-based assay and in PBMCs by real-time PCR. mRNA profiling confirmed the down-regulation of TNFα, IFNγ, IL-10, IL-17, IL-21, and IL-12p40 and the up-regulation of IL-6 in CD3/CD28-stimulated PBMCs following co-culture with DPSCs. IL-12p40 encodes a subunit common to IL-12 and IL-23, which are heterodimeric proteins. The down-regulation of IL-12p40 mRNA can thus explain the decrease of both IL-12 and IL-23 proteins. In resting conditions, mRNA profiling of PBMCs confirmed the up-regulation of IL-6 following co-culture, while IL-10 and TNFα remained unchanged instead of up-regulated. This can be explained by hypothesizing a main production of IL-10 and TNFα by monocytes, which remain partly attached on culture wells and/or DPSCs thus are not fully harvested by centrifugation of culture supernatants.

Since MSCs can promote or counteract inflammation depending on the cytokine milieu in the microenvironment and on the immune cell state, it is difficult to foresee what will happen following the administration of MSCs *in vivo* based on the results *in vitro*. In line with the herein results, patients with COVID-19 treated with MSCs showed lower levels of TNFα (Leng et al., 2020; Zhang et al., 2020), whereas they showed lower levels of IL-6 (Guo et al., 2020; Meng et al., 2020; Shu et al., 2020; Zhang et al., 2020) and higher levels of IL-10 (Leng et al., 2020) in plasma/serum samples.

Herein, data do not allow to understand whether direct contact between DPSCs and PBMCs from COVID-19 patients is needed to have the modulation of cytokines. MSC effects can be mediated by cell surface molecules (e.g., PD-L1 and FasL), acting by cell contact, as well as secreted molecules (e.g., IDO), acting in a paracrine way. Literature data indicate that DPSCs can have comparable regulatory effects on phytohemagglutinin-activated CD3+ T lymphocytes from healthy donors after mixed lymphocyte DPSC cultures and transwell cultures in which the two kinds of cells are separated (Demircan et al., 2011). This supports the existence of soluble immunomodulating factors and fosters investigations on the use of DPSC/MSC-derived secretome instead of whole cells in patients.

The comparison of cytokine concentrations in supernatants of PBMCs from COVID-19 patients and healthy subjects indicated that T lymphocytes from COVID-19 patients have lower ability to produce IL-10 and higher ability to produce IL-18 following activation. The main role of IL-10 is to maintain immune homeostasis. COVID-19 patients have shown higher circulating levels of IL-10 than have healthy subjects (Costela-Ruiz et al., 2020). We can speculate that the production of IL-10 *in vivo* derives mainly from cell types other than T lymphocytes, or T lymphocytes become exhausted regarding IL-10 production or there is an impairment of a specific T cell subset (Brockmann et al., 2018) in COVID-19 patients. IL-18 is a proinflammatory cytokine that promotes Th1 responses, IFNγ production and can be induced by inflammasome activation. IL-18 has been found overexpressed in symptomatic and severe COVID-19 patients vs. healthy controls (Chi et al., 2020; Fraser et al., 2020). Herein data further support a possible role of IL-18 in disease pathogenesis.

## Conclusion

We are aware of the limits of our study: the fact that a low number of samples/patients were analyzed and that only two assays were performed (the quantification of cytokines in conditioned media and mRNA profiling in PBMCs). Results are explorative but indicate that DPSCs can down-regulate the production of cytokines by activated PBMCs, e.g., TNFα, IFNγ, IL-10, and IL-17A, which are up-regulated in COVID-19 patients. This provides a proof of principle for the use of DPSCs/MSCs in COVID-19 and fosters further studies to characterize the effects of MSCs in COVID-19 patients. In particular, impacts of the possible up-regulation of IL-6 and GM-CSF would need attention. Finally, alterations in the production of IL-10 and IL-18 by T lymphocytes might be involved in COVID-19 pathogenesis.

## Data Availability Statement

The original contributions presented in the study are included in the article/Supplementary Material, further inquiries can be directed to the corresponding author/s.

## Ethics Statement

The studies involving human participants were reviewed and approved by the Comitato Etico dell’Area Vasta Emilia Nord, Emilia Romagna, Italy. The patients/participants provided their written informed consent to participate in this study.

## Author Contributions

SC conceived the study, designed and performed the experiments, analyzed the data, interpreted the results, and wrote the manuscript. MB performed the experiments and contributed to the data analysis. GD, MM, NF, AN, GC, and FM recruited the patients. EP and AP obtained and characterized the DPSCs. GC provided the DPSCs and contributed to the experimental design. LB and AZ contributed to the data analysis and interpretation. CS conceived the study and contributed to interpret the results and to writing the manuscript. All authors critically reviewed the manuscript and approved the submitted version.

## Conflict of Interest

The authors declare that the research was conducted in the absence of any commercial or financial relationships that could be construed as a potential conflict of interest.
