# Supplementary material for: Human Dental Pulp Stem Cells Modulate Cytokine Production in vitro by Peripheral Blood Mononuclear Cells From Coronavirus Disease 2019 Patients
Source: Front Cell Dev Biol. 2021 Feb 5;8:609204. doi: 10.3389/fcell.2020.609204 (PMC7901970; doi:10.3389/fcell.2020.609204)
Supplement: Supplementary file 1 [file Data_Sheet_1.doc]

**Figure S1 legend.** **Cytokine gene expression levels in PBMCs from COVID-19 patients following co-culture with DPSCs.** RNA was extracted from PBMC collected by centrifugation of supernatants after 48 hours of culture. RNA was retrotranscribed and specific mRNAs were quantified by real-time PCR using SYBR green based assays. (A) Effects of co-culture in resting conditions. (B) Effects of co-culture with activation of T lymphocytes. Expression normalized over the geometric mean of three housekeeping genes is shown: 2^-ΔCt^. (C) Fold changes of gene expressions by DPSC treated PBMCs over untreated PBMCs from each patient are shown using box-and-whisker graphs with Tukey whiskers. One sample among resting PBMCs was negative for IL-6 expression and two samples among stimulated PBMCs with DPSC contact were negative for IL-12p40 expression. In these cases a Ct = 35 was assigned to draw the graphs.


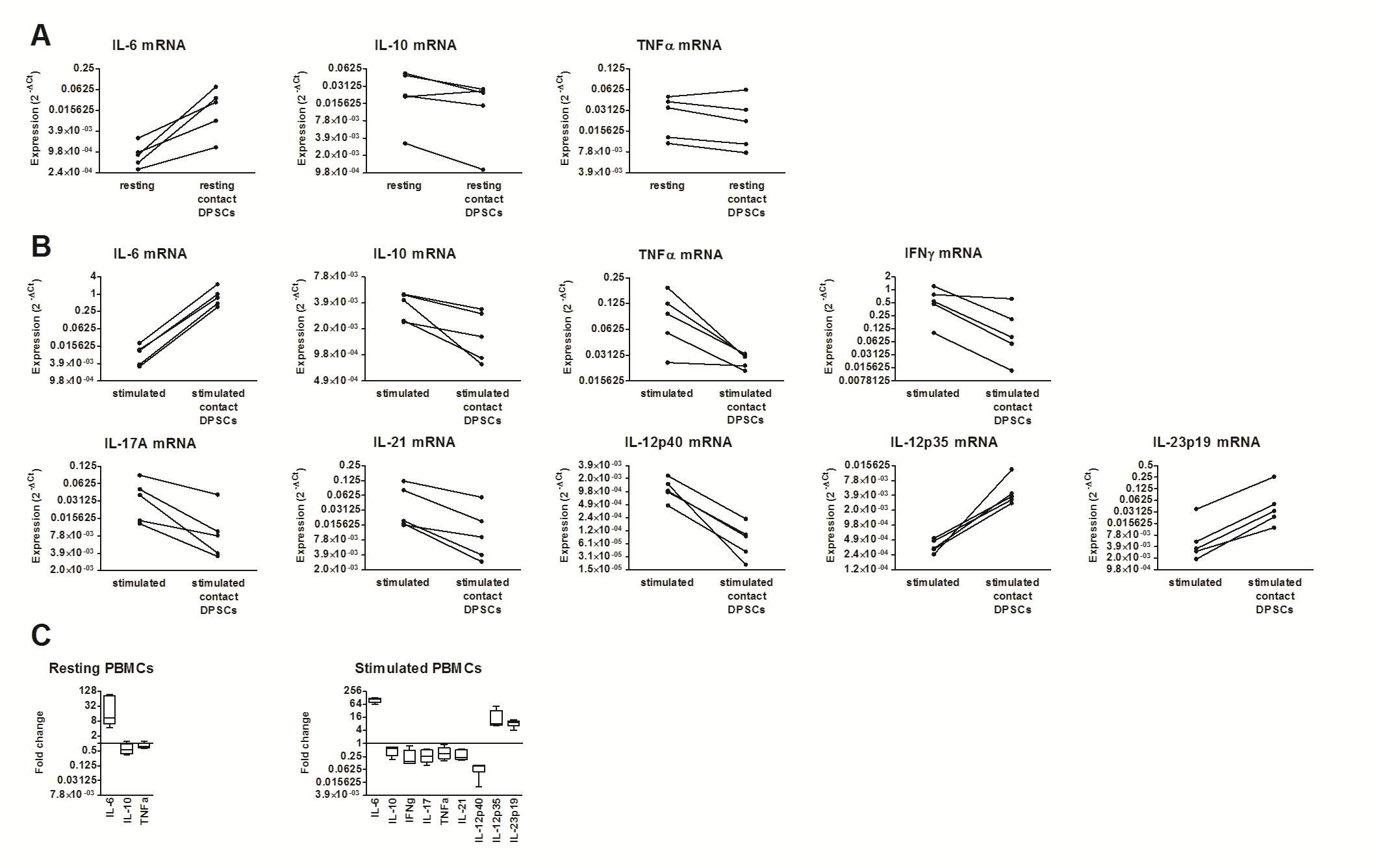


**Table S1.** Cytokine concentrations (pg/ml) detected in supernatants conditioned by PBMCs from COVID-19 patients with and without 48 hour co-culture with DPSCs in resting conditions and T lymphocyte stimulation by ligation of CD3/CD28.

| **PBMC resting** | **GM-CSF** | **IFNγ** | **TNFα** | **IL-10** | **IL-12** | **IL-13** | **IL-17A** | **IL-18** | **IL-1b** | **IL-**  **2** | **IL-21** | **IL-22** | **IL-23** | **IL-27** | **IL-**  **4** | **IL-**  **5** | **IL-**  **6** | **IL-**  **9** |
| --- | --- | --- | --- | --- | --- | --- | --- | --- | --- | --- | --- | --- | --- | --- | --- | --- | --- | --- |
| 1 | < | < | < | 5.8 | < | < | < | < | 5.2 | < | < | < | < | < | < | < | 492 | < |
| 2 | < | < | < | 8.0 | < | < | < | < | < | < | < | < | < | < | < | < | 125 | < |
| 3 | < | < | < | 7.7 | < | < | < | < | < | < | 12.2 | 21.6 | < | < | < | < | 241 | < |
| 4 | < | < | < | 4.7 | < | < | < | < | < | < | < | < | < | 25.9 | < | < | 61 | < |
| 5 | < | < | < | 2.1 | < | < | < | < | < | < | < | < | < | < | < | < | 19 | < |
| 6 | < | < | < | 1.9 | < | < | < | < | < | < | < | < | < | < | < | < | 63 | < |
| 7 | < | < | < | < | < | < | < | < | < | < | < | < | < | < | < | < | < | < |
| 8 | < | < | < | 5.6 | < | < | < | < | < | < | < | < | < | < | < | < | 50 | < |
| 9 | < | < | < | < | < | < | < | < | < | < | < | < | < | < | < | < | 151 | < |
| 1 - DPSC | 46.7 | < | 7.2 | 20.5 | < | < | < | 11.2 | 7.9 | 20.0 | 5.7 | < | < | < | < | 21.1 | 15996 | < |
| 2 - DPSC | 50.6 | < | 7.2 | 46.6 | < | < | < | 12.5 | 5.2 | 22.4 | < | < | < | < | < | 23.2 | 19115 | < |
| 3 - DPSC | 63.4 | < | 7.2 | 40.0 | < | < | < | 18.4 | 3.9 | 26.1 | < | < | < | < | < | 29.3 | 33482 | < |
| 4 - DPSC | 44.7 | < | 7.6 | 13.1 | < | < | 5.3 | 12.4 | 4.0 | 23.4 | 3.7 | < | < | < | < | 22.1 | 13744 | < |
| 5 - DPSC | 36.2 | < | 5.2 | 14.8 | < | < | < | 11.8 | 1.0 | 22.6 | < | < | < | < | < | 24.3 | 11332 | < |
| 6 - DPSC | 45.6 | < | 4.8 | 29.6 | < | < | 1.2 | 8.6 | 3.8 | 20.3 | < | < | < | < | < | 20.6 | 16235 | < |
| 7 - DPSC | 22.6 | < | 5.2 | 7.5 | < | < | < | 13.1 | 1.0 | 13.4 | < | < | < | < | < | 15.3 | 3383 | < |
| 8 - DPSC | 36.2 | < | 7.6 | 31.8 | < | < | 5.8 | 11.8 | 2.6 | 22.6 | < | < | < | < | < | 26.4 | 20550 | < |
| 9 - DPSC | 69.8 | < | 7.0 | 15.1 | < | < | 1.9 | 11.0 | 3.8 | 22.9 | < | < | < | < | < | 22.6 | 25677 | < |
| **PBMC stimulated** | **GM-CSF** | **IFNγ** | **TNFα** | **IL-10** | **IL-12** | **IL-13** | **IL-17A** | **IL-18** | **IL-1b** | **IL-**  **2** | **IL-21** | **IL-22** | **IL-23** | **IL-27** | **IL-**  **4** | **IL-**  **5** | **IL-**  **6** | **IL-**  **9** |
| 1 | 2392 | 4174 | 2182 | 1100 | 40 | 502 | 1187 | 826 | 361 | 4408 | 3227 | 304 | 118 | 94 | 439 | 157 | 3969 | 1228 |
| 2 | 444 | 908 | 1138 | 373 | 8 | 248 | 505 | 319 | 58 | 2330 | 103 | 204 | 55 | 73 | 191 | 115 | 4874 | 389 |
| 3 | 1428 | 2715 | 1793 | 500 | 22 | 739 | 967 | 771 | 102 | 2806 | 135 | 246 | 61 | 85 | 437 | 208 | 6083 | 936 |
| 4 | 8009 | 4797 | 2777 | 1038 | 110 | 1123 | 1935 | 1144 | 310 | 5361 | 7834 | 898 | 436 | 134 | 1133 | 306 | 9273 | 2024 |
| 5 | 1731 | 5234 | 2032 | 834 | 58 | 659 | 895 | 944 | 353 | 4757 | 1907 | 315 | 99 | 85 | 399 | 188 | 4248 | 1493 |
| 6 | 692 | 5196 | 1831 | 937 | 110 | 531 | 391 | 924 | 120 | 2895 | 235 | 368 | 162 | 73 | 263 | 161 | 4401 | 975 |
| 7 | 5576 | 4856 | 2193 | 902 | 37 | 480 | 1809 | 829 | 126 | 4862 | 3061 | 407 | 172 | 87 | 731 | 193 | 2949 | 301 |
| 8 | 983 | 4390 | 1871 | 1083 | 45 | 615 | 912 | 888 | 262 | 3709 | 435 | 556 | 81 | 73 | 315 | 159 | 8646 | 728 |
| 9 | 8761 | 5239 | 2554 | 2166 | 42 | 3024 | 1255 | 1451 | 213 | 6644 | 4839 | 633 | 148 | 103 | 2709 | 610 | 4090 | 5748 |
| 1 - DPSC | 5428 | 1960 | 537 | 851 | 14 | 494 | 753 | 566 | 177 | 3826 | 204 | 257 | 53 | 75 | 633 | 120 | 37916 | 455 |
| 2 - DPSC | 1694 | 181 | 59 | 201 | 3 | 111 | 194 | 119 | 35 | 787 | 26 | 175 | 40 | 28 | 198 | 52 | 24805 | 93 |
| 3 - DPSC | 3870 | 641 | 254 | 324 | 8 | 773 | 517 | 466 | 93 | 1715 | 38 | 193 | 35 | 48 | 454 | 119 | 29180 | 308 |
| 4 - DPSC | 7800 | 5032 | 1717 | 922 | 63 | 1894 | 1622 | 1350 | 350 | 5435 | 2316 | 1231 | 135 | 137 | 1129 | 306 | 28146 | 2365 |
| 5 - DPSC | 6543 | 3099 | 464 | 636 | 24 | 731 | 552 | 798 | 317 | 3238 | 402 | 311 | 61 | 62 | 742 | 133 | 24330 | 995 |
| 6 - DPSC | 3321 | 3622 | 166 | 369 | 44 | 380 | 166 | 646 | 156 | 1173 | 39 | 430 | 45 | 23 | 376 | 77 | 20781 | 267 |
| 7 - DPSC | 4379 | 1856 | 1022 | 540 | 13 | 606 | 1171 | 547 | 136 | 4321 | 695 | 508 | 54 | 55 | 501 | 137 | 23887 | 177 |
| 8 - DPSC | 4758 | 2121 | 302 | 772 | 22 | 520 | 595 | 574 | 255 | 2575 | 94 | 791 | 47 | 44 | 507 | 92 | 24907 | 435 |
| 9 - DPSC | 4654 | 3237 | 1077 | 1003 | 27 | 2657 | 727 | 1097 | 171 | 4008 | 1127 | 376 | 67 | 54 | 1369 | 342 | 30720 | 1960 |

< indicated values lower than the lower limits of detection.

**Table S2.** Cycle threshold values of the investigated cytokines obtained by real-time PCR performed on cDNAs from COVID-19 PBMCs with and without co-culture with DPSCs in resting conditions and T lymphocyte stimulation by ligation of CD3/CD28.

| **PBMC**  **resting** | **GAPDH Ct** | **Actin**  **Ct** | **POLR2A**  **Ct** | **IL-6**  **Ct** | **TNFα**  **Ct** | **IFNγ**  **Ct** | **IL-10**  **Ct** | **IL-17**  **Ct** | **IL-12p35**  **Ct** | **IL-12p40**  **Ct** | **IL-23p19**  **Ct** | **IL-2**  **Ct** |
| --- | --- | --- | --- | --- | --- | --- | --- | --- | --- | --- | --- | --- |
| 1 | 20.2 | 16.1 | 23.5 | 29.7 | 26.3 | nd | 25.2 | nd | nd | nd | nd | nd |
| 2 | 21.1 | 16.2 | 23.6 | 31.1 | 24.6 | nd | 24.4 | nd | nd | nd | nd | nd |
| 3 | 20.5 | 16.1 | 23.8 | 28.5 | 26.2 | nd | 24.1 | nd | nd | nd | nd | nd |
| 6 | 20.5 | 16.3 | 23.3 | 30.1 | 24.1 | nd | 25.4 | nd | nd | nd | nd | nd |
| 7 | 24.2 | 20.2 | 26.0 | neg | 28.2 | nd | 31.6 | nd | nd | nd | nd | nd |
| 1 - DPSC | 20.2 | 16.7 | 23.8 | 27.0 | 27.1 | nd | 26.2 | nd | nd | nd | nd | nd |
| 2 - DPSC | 21.8 | 18.3 | 24.9 | 26.3 | 26.5 | nd | 26.7 | nd | nd | nd | nd | nd |
| 3 - DPSC | 20.2 | 16.8 | 23.9 | 25.3 | 26.7 | nd | 25.5 | nd | nd | nd | nd | nd |
| 6 - DPSC | 19.9 | 16.1 | 23.4 | 23.3 | 23.6 | nd | 24.9 | nd | nd | nd | nd | nd |
| 7 - DPSC | 23.4 | 19.6 | 26.0 | 32.4 | 28.4 | nd | 32.7 | nd | nd | nd | nd | nd |
| **PBMC stimulated** |  |  |  |  |  |  |  |  |  |  |  |  |
| 1 | 16.4 | 14.8 | 21.8 | 25.5 | 21.6 | 18.3 | 26.2 | 23.5 | 29.0 | 28.5 | 26.5 | 21.1 |
| 2 | 17.1 | 15.0 | 21.8 | 24.3 | 21.2 | 21.1 | 25.4 | 22.4 | 28.7 | 27.7 | 26.2 | 23.7 |
| 3 | 16.9 | 14.7 | 21.7 | 24.0 | 20.6 | 18.7 | 25.3 | 21.9 | 28.6 | 27.6 | 25.7 | 23.3 |
| 6 | 16.0 | 14.2 | 21.6 | 25.4 | 22.3 | 17.4 | 25.7 | 23.3 | 28.6 | 26.4 | 26.2 | 23.0 |
| 7 | 18.1 | 15.4 | 22.8 | 24.2 | 21.0 | 18.3 | 26.5 | 22.1 | 30.5 | 27.3 | 25.1 | 21.6 |
| 1 - DPSC | 18.2 | 15.8 | 22.2 | 19.6 | 24.2 | 22.2 | 27.8 | 25.5 | 27.1 | 33.1 | 24.0 | 24.3 |
| 2 - DPSC | 19.1 | 16.1 | 23.2 | 19.2 | 24.2 | 25.5 | 27.5 | 27.3 | 27.3 | 32.7 | 25.6 | 27.7 |
| 3 - DPSC | 18.6 | 16.2 | 23.0 | 19.5 | 24.1 | 23.2 | 27.5 | 25.8 | 27.3 | 32.4 | 24.0 | 27.1 |
| 6 - DPSC | 18.4 | 16.3 | 22.9 | 20.5 | 24.4 | 19.7 | 29.1 | 27.2 | 26.9 | neg | 24.9 | 25.8 |
| 7 - DPSC | 22.2 | 20.3 | 26.7 | 21.7 | 28.0 | 25.2 | 33.3 | 27.5 | 29.1 | neg | 26.3 | 27.0 |

nd = not determined

neg = negative

**Table S3.** Post hoc analysis of the fold changes of cytokine levels in conditioned media to compute the achieved power.

|  | **GM-CSF** | **IFNγ** | **TNFα** | **IL-10** | **IL-12** | **IL-13** | **IL-17A** | **IL-18** | **IL-1β** | **IL-2** | **IL-21** | **IL-22** | **IL-23** | **IL-27** | **IL-4** | **IL-5** | **IL-6** | **IL-9** |
| --- | --- | --- | --- | --- | --- | --- | --- | --- | --- | --- | --- | --- | --- | --- | --- | --- | --- | --- |
| **Effect size d** | 1.02 | 1.92 | 3.91 | 2.13 | 4.81 | 0.03 | 3.24 | 1.30 | 0.35 | 1.38 | 13,1 | 0.11 | 3.32 | 1.86 | 0.38 | 2.11 | 1.76 | 1.58 |
| **Power** | 0.74 | 0.99 | 1.00 | 0.99 | 1.00 | 0.05 | 1.00 | 0.91 | 0.15 | 0.94 | 1.00 | 0.06 | 1.00 | 0.99 | 0.16 | 0.99 | 0.99 | 0.98 |

**Table S4.** Cytokine concentrations (pg/ml) detected in supernatants conditioned for 48 hours by PBMCs from healthy controls in resting conditions and stimulated by ligation of CD3/CD28.

| **PBMC**  **resting** | **GM-CSF** | **IFNg** | **TNFa** | **IL-10** | **IL-12** | **IL-13** | **IL-17A** | **IL-18** | **IL-1b** | **IL-2** | **IL-21** | **IL-22** | **IL-23** | **IL-27** | **IL-4** | **IL-5** | **IL-6** | **IL-9** |
| --- | --- | --- | --- | --- | --- | --- | --- | --- | --- | --- | --- | --- | --- | --- | --- | --- | --- | --- |
| 1 HC | < | < | < | 3.7 | < | < | < | < | 2.8 | < | < | < | < | < | < | < | 23 | < |
| 2 HC | < | < | < | 11.6 | < | < | < | < | 6.7 | < | < | < | < | < | < | < | 399 | < |
| 3 HC | < | < | < | 5.3 | < | < | < | < | 3.0 | < | < | < | < | < | < | < | 168 | < |
| 4 HC | < | < | < | 2.8 | < | < | < | < | < | < | < | < | < | < | < | < | 12 | < |
| 5 HC | < | < | < | < | < | < | < | < | < | < | < | < | < | < | < | < | 20 | < |
| 6 HC | < | < | < | 3.3 | < | < | < | < | 1.5 | < | < | < | < | < | < | < | 124 | < |
| 7 HC | < | < | < | 17.5 | < | < | < | < | 15.0 | < | < | < | < | < | < | < | 1625 | < |
| 8 HC | < | < | < | 4.6 | < | < | < | < | < | < | < | < | < | < | < | < | 93 | < |
| 9 HC | < | < | < | < | < | < | < | < | < | < | < | < | < | < | < | < | 101 | < |
| **PBMC stimulated** |  |  |  |  |  |  |  |  |  |  |  |  |  |  |  |  |  |  |
| 1 HC | 3990 | 2953 | 2233 | 2593 | 69 | 583 | 1458 | 568 | 139 | 5539 | 4562 | 348 | 69 | 97 | 696 | 202 | 3536 | 1317 |
| 2 HC | 1155 | 2411 | 1003 | 1753 | 39 | 842 | 698 | 550 | 65 | 1468 | 329 | 361 | 38 | 60 | 452 | 226 | 1785 | 1893 |
| 3 HC | 1476 | 3475 | 1966 | 3248 | 32 | 416 | 1692 | 564 | 157 | 3946 | 1758 | 436 | 51 | 104 | 368 | 149 | 3444 | 896 |
| 4 HC | 3148 | 5000 | 2458 | 3950 | 110 | 566 | 1142 | 886 | 122 | 5240 | 3474 | 550 | 192 | 109 | 548 | 168 | 3402 | 883 |
| 5 HC | 2332 | 9010 | 3830 | 4016 | 290 | 235 | 744 | 489 | 84 | 9202 | 8687 | 378 | 167 | 68 | 119 | 69 | 4562 | 280 |
| 6 HC | 3477 | 7680 | 5562 | 11049 | 249 | 614 | 1363 | 502 | 110 | 5241 | 7991 | 1252 | 168 | 122 | 312 | 95 | 7220 | 1269 |
| 7 HC | 729 | 6143 | 1643 | 2911 | 98 | 673 | 466 | 476 | 48 | 1463 | 954 | 713 | 80 | 71 | 171 | 182 | 3810 | 1357 |
| 8 HC | 2799 | 10795 | 5483 | 14629 | 115 | 509 | 1253 | 563 | 111 | 5536 | 4143 | 1164 | 138 | 94 | 154 | 95 | 5671 | 864 |
| 9 HC | 1010 | 7849 | 2935 | 5073 | 67 | 216 | 537 | 435 | 66 | 4569 | 2547 | 507 | 141 | 66 | 76 | 41 | 3532 | 198 |

< indicated values lower than the lower limits of detection
